# Improving Reproductive Performance and Health of Mammals Using Honeybee Products

**DOI:** 10.3390/antiox10030336

**Published:** 2021-02-24

**Authors:** Nesrein M. Hashem, Eman M. Hassanein, Jesus Simal-Gandara

**Affiliations:** 1 Department of Animal and Fish Production, Faculty of Agriculture (El-Shatby), Alexandria University, Alexandria 21545, Egypt; em.mostafa@alexu.edu.eg; 2 Nutrition and Bromatology Group, Department of Analytical Chemistry and Food Science, Faculty of Food Science and Technology, University of Vigo-Ourense Campus, E-32004 Ourense, Spain

**Keywords:** reproduction, mammals, honeybee products, active compounds

## Abstract

Honeybee products have positive effects on the reproductive performance of mammals. Many honeybee product constituents are biologically active, with antioxidant, antimicrobial, antiviral, anti-inflammatory, immunomodulatory, antifungal, wound-healing, and cardio-protective properties. Honeybee products also improve male and female fertility rates by enhancing gamete cryopreservation, in vitro maturation and fertilization, and embryo development. Previously published studies confirmed their efficacy for alleviating reproductive toxicity caused by contaminants and lifestyle habits that impair overall health and well-being. However, high-dose oral administration of honeybee products may adversely affect the reproductive system, and unfavorable effects were alleviated by treatment cessation. For this reason, this review proposes that bioactive components from bee products can be used as a strategy for improving the reproductive performance and health of mammals.

## 1. Introduction

Procreation is a pivotal innate physiological event for all creatures. For humankind, reproductive health and fertility are of particular importance for maintaining social, mental, and physical health status. Supporting adequate reproductive performance in food-producing animals is also important to humans, enabling mass production to maintain food security. However, reproductive events and fertility of individuals/organisms are greatly impacted by modern lifestyle circumstances such as increased exposure to environmental and behavioral stresses. According to a World Health Organization report, 60–80 million couples, representing 8–12% of couples worldwide, are currently experiencing infertility [1,2,3]. Similarly, poor reproductive efficiency is evoked in production animals owing to management practices for producing animal products in massive quantities [4]. Therefore, research endeavors to improve and maintain adequate reproductive health are essential for humankind and animals.

The use of natural products as alternatives to synthetic prophylactic and therapeutic drugs is increasingly recommended to improve many aspects of human and animal health [5]. Honeybee products, including honey, propolis, royal jelly, bee pollen, beeswax, drone brood, bee venom, and bee bread, contain several natural bioactive components with various pharmaceutical and nutritional properties. Given the chemical constitutes of these products, honeybee products may have beneficial prophylactic and therapeutic effects on reproductive health in mammals. For example, honey is an energy-rich product that contains substantial concentrations of polyphenolic compounds with antioxidant activity [6,7,8,9], which can improve reproductive events by improving energy status [10,11] and/or redox status [12]. Additionally, propolis has many pharmaceutical properties thanks to its enrichment with flavonoids, which are polyphenolic compounds required for maintaining reproductive health, particularly under stressful conditions such as heat stress [13,14]. Furthermore, products such as royal jelly and drone brood contain sex hormones, and thus can be used to modulate endocrine system functions [15]. Many studies have shown the positive effects of honeybee products on reproductive health in humans [16,17] and animals [14,16,17,18,19,20,21,22,23,24,25,26,27,28,29,30,31,32,33,34,35,36]. However, the potential hazards of honeybee products have also been discussed [37,38]. This review describes honeybee products, emphasizing the unique chemical constituents of each product and their effects on reproductive performance in humans and animals. This will help in exploring the treasure of the natural active compounds in these products, allowing opportunities to detect novel pharmaceutical molecules for safe reproductive health manipulation.

## 2. Honeybee Species and Bioactive Components of Honeybee Products

There are approximately 20,000 known species of bees. Honeybees represent a small portion of all bees, with eight recognized species (*Apis mellifera*, *A. mellifera*; *Apis cerana*, *A. cerana*; *Apis dorsata*, *A. dorsata*; *Apis florea*, *A. florea*; *Apis andreniformis*, *A. andreniformis*; *Apis laboriosa*, *A. laboriosa*; *Apis koschevnikovi*, *A. koschevnikovi*; and *Apis nigrocincta*, *A. nigrocincta)* and 43 subspecies (Table 1). Among these species, *A. mellifera* and *A. cerana* are domesticated by humans, while the other species are wild. In Europe and America, *A. mellifera* (Western honeybee) is the species universally managed by beekeepers. This species has several subspecies, including *A. mellifera ligustica* (Italian bee), *A. mellifera mellifera* (European dark bee), and *A. mellifera carnica* (Carniolan honeybee). *A. cerana* (Asiatic honeybee) is the common species bred for honey production in the tropics [1,2,39,40].

Apitherapy, a branch of alternative medicine that uses honeybee products, has been applied to protect from and treat diseases for many centuries [10,12]. Nevertheless, the physical properties and chemical compositions of honeybee products are highly varied and depend on factors such as plant type, climatic conditions, and geographical region. Moreover, the metabolites, physiology, endogenous enzymes, and flora of the collecting insects can affect the physical properties and chemical composition of honeybee products [39,40]. In the following section, different honeybee products and their major and minor chemical constitutes will be shown.

### 2.1. Honey

Honey is a sweet substance produced by honeybees from collected flower nectar and plant secretions, which are then combined with specific bee substances, deposited, dehydrated, and stored in honeycombs to ripen [13]. Although many honeybee species, wasps, and ants produce different types of honey [4], the legal definition of honey according to the European Union Council [41] is honey that is produced by *A. mellifera* honeybees [4,13].

Chemically, honey is a natural food substance mainly composed of simple sugars, along with minor constituents such as minerals, vitamins, amino acids, organic acids, flavonoids and other phenolic compounds, and aromatic substances [13]. Generally, sugars comprise 95–99% of honey dry matter, mainly in the form of fructose (32–38% of total sugars). In addition, several other monosaccharides (glucose), disaccharides (sucrose and maltose), and oligosaccharides (maltotriose and panose) are found in honey. Low concentrations of proteins (0.5%) in the form of enzymes (amylase, sucrase or α-glucosidase, and glucose oxidase) and more than 20 individual amino acids (proline is the most important) have also been identified [8]. Organic acids (0.57%; gluconic, acetic, butyric, citric, formic, lactic, malic, pyroglutamic, and succinic acids) as well as major and minor minerals (calcium, magnesium, sodium, potassium, phosphorus, sulfur, zinc, iron, copper, and manganese) are also components of honey. In addition, vitamins such as ascorbic acid (vitamin C), thiamine (vitamin B_1_), riboflavin (vitamin B_2_), nicotinic acid (vitamin B_3_), pantothenic acid (vitamin B_5_), pyridoxine (vitamin B_6_), biotin (vitamin B_8_), folic acid (vitamin B_9_), and cyanocobalamin (vitamin B_12_) are present in measurable concentrations [40,42]. Honey also contains significant amounts of bioactive polyphenols, including phenolic acids (vanillic, caffeic, syringic, p-coumaric, ferulic, ellagic, 3-hydroxybenzoic, chlorogenic, 4-hydroxybenzoic, rosmarinic, gallic, and benzoic acids) and flavonoids (quercetin, kaempferol, myricetin, pinobanksin, pinocembrin, chrysin, galangin, hesperetin, and others) [8,43]. The unique chemical composition of honey confers several nutritional and medicinal properties [42]. Honey has a long history of use in traditional medicine owing to its antioxidant, hepato-protective, cardio-protective, anti-inflammatory, antidiabetic, hypolipidemic, anticancer, gastrointestinal-protective, and wound-healing properties. In addition, honey produces antibacterial effects against several microorganisms, including *Escherichia coli*, *Shigella* spp., *Helicobacter pylori*, and *Salmonella* spp. [13].

### 2.2. Royal Jelly

Royal jelly is a thick, viscous, and milky natural substance that is formed as a result of incomplete digestion of honeydew in the stomach of a bee worker [44]. In the hive, royal jelly is fed to larvae developing into female workers and male drones until they are three days old, while individuals developing into future queens are fed royal jelly throughout the larval period. Moreover, royal jelly is provided as a special nutrient for adult queens throughout their lives [13,45,46,47]. Nutritionally, royal jelly is a high-value nutrient, consisting of proteins (27%–41%), carbohydrates (30%), and lipids (3%–19%). It also contains a unique group of nine soluble proteins called major royal jelly proteins (MRJPs), of which MRJPs 1–5 account for approximately 82%. Several antioxidant peptides have been isolated from royal jelly hydrolysate, some with robust hydroxyl radical scavenging activity. Moreover, royal jelly peptides such as jelleines, royalisin, and apisimin exhibit antimicrobial activity [15]. Fatty acids are the major lipid constituents (mainly hydroxydecanoic acid), followed by phenolic lipids, waxes, steroids, and finally phospholipids [48]. Royal jelly also provides a valuable energy source thanks to its enrichment with carbohydrates (3.4%–7.7% glucose, 2.3%–7.8% fructose) and energy-rich molecules such as adenosine monophosphate [15]. In addition, royal jelly contains phytosterols, flavonoids, vitamins (B_9_, B_1_, niacin, B_3_, B_5_, B_2_, B_6_, and beta-carotene), minerals (iron, potassium, zinc, magnesium, and copper), and hormones (prolactin, testosterone, estradiol, and progesterone) [15]. The diverse royal jelly chemical composition confers several pharmaceutical and nutraceutical properties, such as antibacterial, antioxidant, anti-inflammatory, antidiabetic, hypotensive, hepato-protective, antitumor, and hypoglycemic properties [46,47,48,49]. Moreover, immunomodulatory activities and estrogen-like effects have been reported. Accordingly, royal jelly has been widely used in commercial medical products, health foods, and cosmetics for more than 35 years [49].

### 2.3. Propolis

Propolis (bee glue) is a dark-colored resinous substance that honeybees produce by mixing collected plant parts (buds, floral buds, leaves, branches, and barks) and salivary gland secretions. In ancient Greek, *propolis* means defense of the city; the antiseptic and antimicrobial substance is used in the hive to maintain colony health. Thus, propolis has been identified as a natural antibiotic candidate [50]. Raw propolis is typically composed of 50% plant resin, 30% wax, 10% essential and aromatic oil, 5% pollen, and 5% other organic substances. The color of propolis varies from green to brown and reddish, depending on its botanical source [1]. Propolis cannot be commercialized as a raw material; it must be purified and dewaxed via solvent extraction to remove inert materials and preserve the phenolic fractions [13]. Propolis has been classified into seven main types according to the plant source: (1) poplar propolis: the most widespread type of propolis (Europe, North America, New Zealand, and non-tropical regions of Asia), (2) green propolis or Baccharis propolis, (3) red propolis or Clusia (Brazil, Cuba, Venezuela, and Mexico), (4) eucalyptus propolis, (5) Taiwanese green propolis or Macaranga propolis (Okinawa prefecture in Japan, Taiwan, and Indonesia), (6) birch propolis (Russia), and (7) Mediterranean propolis (Greece, Sicily, and Malta) [13]. Among the types of propolis, poplar and Brazil green propolis are the most commercially available and widely studied [50,51]. The biological activities of propolis, including antioxidant, antimicrobial, antiviral, anti-inflammatory, antifungal, wound-healing, and cardio-protective properties, have been ascribed to the action of phenolic compounds and terpenoids [11,14].

### 2.4. Bee Venom

Bee venom, also known as “apitoxin”, is a colorless, acidic liquid that is synthesized in the honeybee venom gland. Honeybee defense mechanisms against threatening attacks involve the injection of bee venom into the body of the attacker, evoking several neurological, immunological, and inflammatory responses [52,53]. The chemical constituents that distinguish bee venom are a group of amphipathic polycationic peptides, predominantly melittin and apamin. Bee venom also contains peptides such as mast-cell degranulating peptide, adolapin, tertiapin, secapin, melittin F, and cardiopep [54], as well as amines such as histamine and catecholamines [55]. Some of these peptides trigger cell lysis, while others act as neurotoxins [56]. Melittin acts as a detergent, binding and breaking down the cell membrane lipid bilayers [57]. Apamin exerts a highly specific toxicity mechanism, blocking the small conductance Ca^2+^-dependent K^+^ channels (SK channels) expressed in the central nervous system, cardiovascular system, and smooth muscle [7]. Despite the toxicity, bee venom has been used as an analgesic, antimutagenic, antinociceptive, and radio-protective immunomodulatory agent and was identified as a potential Parkinson’s disease therapy [7]. Furthermore, some substances extracted from bee venom can be included in domestic animal diets as antimicrobial agents to enhance productive performance and health status [36].

### 2.5. Bee Pollen

Bee pollen is a mixture of flower pollen (male germ element), nectar, enzymes, honey, beeswax, and honeybee salivary secretions gathered and produced by worker honeybees [13]. Bee pollen is a rich source of nutrients such as protein (25%), essential amino acids, oil (6%), polyunsaturated fatty acids (13% linoleic acid and 39% linolenic acid), 28 minerals, 12 vitamins, 11 enzymes or coenzymes, carbohydrates (35%–61%; sucrose, glucose, and fructose), carotenoids, flavonoids, and phytosterols [58,59]. Bee pollen is an energy-boosting food that is used by humans as a diet supplement. The high content of protein, fat, and minerals (particularly Ca, Mg, Fe, and P) in bee pollen provides a nutritional value similar to that of dried legumes. B_5_ and B_3_, vitamin C, and B_2_ levels are comparable to those of beef, vegetables (lettuce and tomatoes), and skimmed milk powder, respectively [60]. Bee pollen is used in complementary and alternative medicine to cure prostatitis, stomach ulcers, and infectious diseases, as well as to prevent and treat high-altitude-sickness syndrome. A wide range of therapeutic properties have been reported, including antimicrobial, antioxidant, hepato-protective, chemo-preventive and anti-carcinogenic, anti-atherosclerotic, anti-inflammatory, antiallergenic, and immunomodulatory activities [60,61,62].

### 2.6. Drone Brood, Beeswax, and Bee Bread

Drone brood (apilarnil) is a lesser-known honeybee product. Drone brood is a milky, yellowish-gray color that consists of the dried powder from 3–7-day-old drone larvae collected from drone cells [63]. Drone brood contains approximately 25%–35% dry matter, 9%–12% protein, 6%–10% carbohydrate, 5%–8% lipid, 2% ash, vitamins (A, B_1_, B_6_, and choline), and minerals (Ca, P, Na, Zn, Mn, Fe, Cu, and K) [64].

Beeswax is a substance produced by honeybee wax gland complexes to make combs; the greatest quantity is produced during the colony growth phase in late spring [65]. Chemically, beeswax contains more than 300 identified components. Hydrocarbons such as heptacosane, nonacosane, hentriacontane, pentacosane, and tricosane are the main components, along with free fatty acids and fatty alcohols, linear wax monoesters, hydroxymonoesters, complex wax esters, and more than 50 other aromatic components [66]. Beeswax is used as an additive in a variety of industrial, pharmaceutical, and cosmetic products; however, to the best of our knowledge, no specific studies on the relationship between beeswax and reproduction have been published [13].

Bee bread contains abundant high-quality proteins; carbohydrates; fatty acids; vitamins (B-group, C, K, E, and D), especially vitamin P (rutin) and provitamin A (carotene); various minerals; oligo-elements (especially K and Fe); essential oils; enzymes; pigments; and other biologically active natural substances [67]. No other natural product contains such high concentrations of vitamin P (rutin; 13 mg/100 g of bee bread), which can improve the condition of blood vessels. Moreover, bee bread boosts the immune system and has been used to tackle chronic inflammation of the prostate, male sterility and impotence, endocrine glands disorders, and decreased libido, as well as to improve intra-uterine nutrition. Bee bread also produces a strong antiseptic effect on a wide range of pathogenic microorganisms [67]. A summary of the chemical composition of honeybee products is shown in Table 2.

## 3. Honeybee Products and Reproductive Health

### 3.1. Biological Effects of Honeybee Products on Reproduction

**Figure 1:**
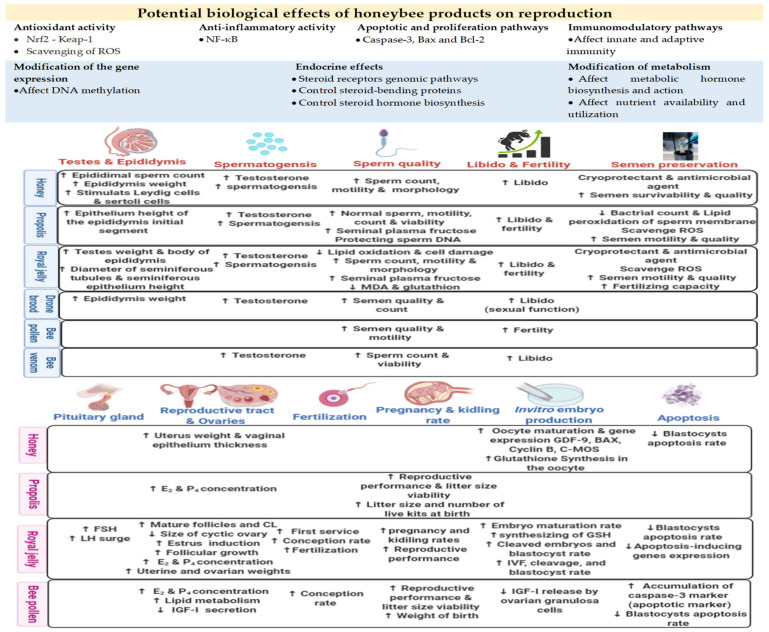
Potential effects of honeybee products on reproductive functions and assisted reproductive techniques (ARTs) in mammals. BAX: bcl-2-like protein 4, Bcl-2: B-cell lymphoma 2, C-MOS: complementary metal–oxide–semiconductor, E_2_: estradiol-17β, FSH: follicle stimulating hormone, GDF-9: growth differentiation factor 9, IGF-I: insulin-like growth factor I, LH: luteinizing hormone, Nrf2-Keap-1: nuclear factor erythroid 2-related factor 2-Keap-1, NF-κB: nuclear factor kappa-light-chain-enhancer of activated B cells, P_4_: progesterone, ROS: reactive oxygen species.

Owing to the diversity of chemical constituents in honeybee products, a wide range of biological effects on reproductive functions could be achieved via several biological mechanisms/pathways. For example, most honeybee products contain phenolic compounds, widely known to affect reproduction in mammals. Phenolic compounds can regulate gonadal steroidogenesis and the functionality and metabolism of sex steroid hormones [70,71]. In addition, phenolic compounds can affect the expression of genes and activity of enzymes (aromatase, topoisomerases I and II, and extracellular signal-regulated kinases) involved in the regulation of reproductive events. Phenolic compounds can also contribute to cellular apoptotic/proliferation, epigenetic, antioxidant, and inflammatory pathways [72]. Metabolic status may also be affected by phenols owing to their ability to regulate metabolic hormone signals such as growth hormone, insulin-like growth factors, and triiodothyronine, as well as lipid, protein, and carbohydrate metabolism. Furthermore, sex hormones have been detected in some bee products, such as royal jelly and drone brood, which can thus modulate endocrine system functions [15]. Drone brood is rich in male sex hormones, especially testosterone, producing an androgenic effect that enhances male sex features. Drone brood can also be classified as a natural anabolic agent that can increase body muscle weight in male individuals [63,73]. Additionally, nutrients, vitamins, and minerals can directly affect reproductive tissues, reinforcing their functions and improving gametogenesis and/or the quality of gametes [74]. Therefore, honeybee products can provide effective tools for improving reproductive functions in vivo and assisted reproductive technique (ART) outputs in vitro. The potential effects of honeybee products on mammalian reproductive functions and ARTs are shown in Figure 1.

### 3.2. Applications for Improving Male Fertility

Recent studies on the effects of honeybee products on male reproductive performance in mammalian species are shown in Table 3. Several studies have supported the traditional use of honey as a natural product to enhance reproductive efficiency and fertility in males [31,32,33]. Honey has been demonstrated to improve libido, erectile function, spermatogenesis, epididymal sperm count, and normal sperm percentage, and reduce the percentage of sperm head and tail abnormalities and chromatin damage in mammalian species, including humans [16,17] and rats [31,32,33]. The positive effects of honey on male reproductive performance are attributed to several different mechanisms. Honey can increase the activity of enzymes that affect sperm quality, such as sorbitol dehydrogenase [31], which converts sorbitol into fructose, an important nutrient for sperm metabolism and motility. Additionally, honey administration improves spermatogenesis by enhancing male sex hormones, specifically testosterone. This enhancement was mediated by luteinizing hormone synthesis, Leydig cell viability, upregulation of steroidogenic acute regulatory protein expression, and aromatase activity in the testes [75]. In addition, bioactive compounds with antioxidant activity (e.g., antioxidant polyphenols such as flavonoids and phenolic acids) can provide reproductive organs with a robust defense system against oxidative stress induced by elevated levels of reactive oxygen species (ROS) [33]. Furthermore, honey can treat dysfunctional erection or impotence by modulating the biosynthesis of nitric oxide, a chemical substance involved in vasodilatation that affects erectile function [16,17].

Propolis is another honeybee product shown to improve the reproductive performance of mammalian males. Several studies confirmed that administration of propolis improved sperm count and testis, seminal vesicle, and epididymis weights, and reduced the percentage of sperm head and tail abnormalities [14,19,20,21,22,23]. Moreover, the administration of propolis extract increased serum testosterone levels, coupled with increased activity and expression of testicular steroidogenesis enzymes including 3β-hydroxysteroid dehydrogenase (3β-HSD) and 17β-hydroxysteroid dehydrogenase (17β-HSD) [24], improving testosterone levels [25]. Propolis also shows strong antioxidant and modulatory activity on cellular mitochondrial energy production [19].

Royal jelly also produced many beneficial effects on male reproductive performance in mammals. As indicated by studies conducted on adult male rats [23,26,27,28] and rabbits [22,29,30], royal jelly enhanced spermatogenesis, steroidogenesis, sperm quality traits, and libido of treated males. Interestingly, royal jelly contains acetylcholine (1 mg/g dry weight), a peripheral and central neurotransmitter. Acetylcholine can stimulate gonadotropin secretion at both hypothalamic and hypophyseal levels, consequently increasing plasma testosterone levels. Most studies have focused on the major products of honeybees (honey, propolis, and royal jelly); however, beneficial effects of other honeybee products, such as drone brood [34,35], bee venom [36], and bee pollen [18], on male reproductive health have also been reported. These products contain active constituents that can affect reproductive functions, such as apistimul in drone brood [34].

### 3.3. Applications for Improving Female Fertility

Table 4 summarizes recent studies on the effects of honeybee products on female reproductive performance in mammalian species. Honey [76], royal jelly [46,77,78,79,80,81], propolis [82,83], and bee pollen [82,83,84,85] were demonstrated to improve female reproductive performance in several mammals. Treatment with royal jelly improved estrus response and pregnancy rate in ewes by enhancing ovarian follicle growth and development and estradiol secretion [80]. Interestingly, Gimenez-Diaz [78] and Kridli [86] reported that royal jelly can mimic the actions of gonadotropins to improve estrus response and conception rate in ewes, providing a promising natural alternative to equine chorionic gonadotropin (eCG). The positive effects of royal jelly on reproductive performance may be partially ascribed to its estrogenic effects [81].

The positive effects of bee pollen on female reproductive performance are well-documented. Bee pollen can improve rabbit doe reproductive performance and milk production and enhance the immune status and growth performance of their offspring [84,85,86]. These results were attributed to the high micronutrient content (e.g., polyunsaturated fatty acids, minerals, vitamins, amino acids) and biological actions of flavonoids, carotenoids, and phenolic compounds in bee pollen [81]. Furthermore, bee pollen can regulate sex steroidogenesis, specifically progesterone and estradiol [87,88]. Hormonal balance is important for adjusting the mechanisms underlying the fate of ovarian follicles, involving cross-dialog between pro-apoptotic (caspase-3, Bax) and pro-survival anti-apoptotic (Bcl-2) molecules [87,88]. The dose and time of administration of bee pollen can both affect these pathways; positive and negative effects on ovarian follicle development and growth could be obtained using different administration methods [87].

In models designed to study menopausal symptoms, Zaid et al. [76] noticed that daily consumption of Tualang honey for two weeks by ovariectomized female rats suppressed menopause-related reproductive disorders, including uterine atrophy, vaginal epithelium atrophy, and osteoporosis. The positive effects of Tualang honey are likely due to flavonoids, particularly kaempferol and quercetin, which have weak estrogenic activity. Further, flavonoids are strong free radical scavengers, protecting organisms from the destructive actions of ROS [72,76]. Interestingly, local vaginal application of honey and royal jelly before sexual intercourse improved fertility in couples having trouble conceiving [88].

## 4. Honeybee Products and Assisted Reproductive Techniques

### 4.1. Male-Associated Assisted Reproductive Techniques

The efficiency of ARTs depends on the appropriate manipulation of gametes (sperm cells) during each stage of handling, starting from semen collection, followed by dilution, cooling, cryopreservation, and finally transfer to the female reproductive tract. During these processes, sperm cells might be exposed to thermal and physicochemical stresses, which can cause serious damage to the sperm cell membrane and DNA, thereby decreasing semen quality [89,90].

One of the important factors that can affect sperm cell quality is the medium in which sperm cells are placed for ART. Ideal media should minimize and protect sperm cells against physicochemical stresses; thus, the composition should prevent alterations to the structure and function of sperm cells [90]. Several studies have described the benefits of honey as a natural cryoprotectant agent for maintaining semen quality kinetic parameters, sperm cell membrane and DNA integrity, and sperm cell morphology when included in semen cryopreservation media (human [91], cattle bull semen [92,93], buffalo bull semen [94], and rabbits [95,96]) and liquid storage media [97].

Honey contains a mixture of sugars, proteins, enzymes, amino acids and organic acids, vitamins, phenolic acids, and flavonoids that confer high antioxidant activity. An appropriate mixture of nutrients and bioactive components is necessary for maintaining adequate sperm quality. For example, simple sugars (glucose, fructose, and sucrose) in honey provide sperm cells with a preferable energy source [94,98,99,100]. Additionally, the presence of components, such as organic acids (gluconic acid), phenolic acids, and flavonoids, with antioxidant and antibacterial activities can play a crucial protective role against the harmful effects of ROS and microbial attack [32]. Thus far, inhibitory effects of honey on 60 species of gram-positive and gram-negative bacteria (*Staphylococcus aureus, Escherichia coli, Staphylococcus epidermidis,* and *Bacillus cereus*) have been reported; these effects are comparable in strength to antibiotics penicillin, streptomycin, and kanamycin [101].

Other studies have reported the beneficial effects of including royal jelly in sperm cell-processing media on sperm cell quality and, later, fertility in some mammals (rams [102,103], goats [104], and buffalo bull [105,106]). The protective role of royal jelly was mainly ascribed to its unique amino acid profile [107]. Royal jelly contains many vital amino acids, including valine, aspartic acid, isoleucine, tyrosine, glycine, lysine, proline, cysteine, and leucine. Proline has been demonstrated to maintain sperm cell membrane integrity under stressful conditions [108] and both cysteine and proline act as robust antioxidant agents to eliminate ROS and stimulate glutathione enzyme synthesis and activity during semen cooling–freezing processes [108]. Moreover, royal jelly vitamins (C and E) and 10-hydroxy-2-decenoic acid provide protective effects to the sperm cell membrane [109,110]. However, high concentrations of royal jelly produced negative impacts on sperm cell quality [103], thus careful consideration of the concentration in media is essential.

Propolis has also been included in semen preservation media, providing antioxidant and antimicrobial activity [111,112,113] and maintaining semen characteristics such as motility, viability, and DNA integrity [114]. The effects of honeybee products on male ARTs are shown in Table 5.

### 4.2. Female-Associated Assisted Reproductive Techniques

For females (Table 6), in vitro maturation (IVM) and in vitro fertilization (IVF) are the most common ARTs, the outcomes of which relate to oocyte quality. Thus, improving the oocyte microenvironment during IVM and IVF has the potential to improve ART outcomes, with corresponding improvements in reproductive efficiency in humans and animals. Under normal circumstances, oocytes receive essential elements, proteins, growth factors, hormones, antioxidants, and other compounds directly from the female reproductive fluids. Consequently, artificially formulated media for oocyte IVM or IVF should contain elements that correspond to the natural sources in the fluids of the female reproductive tract [115,116]. Given the chemical constituents of honeybee products, the inclusion of the honeybee product(s) in IVM or IVF media may have positive effects on oocyte maturation, oocyte fertilizability, and division and embryo development. The addition of honey (black seed bee honey) to sheep IVM medium has been shown to improve oocyte maturation rate, glutathione levels, and gene expression of GDF-9, BAX, cyclin B, C-MOS, and IGF1 genes. Similarly, the addition of royal jelly to goat oocyte [117] medium or ovine IVM medium [118] improved oocyte and nuclear maturation rate, fertilization rate, and blastocyst formation. These improvements seem to be a result of decreased expression of apoptosis-inducing genes, increased glutathione-S-transferase enzymes content, and enhanced mitochondrial activity, which lead to a reduction in blastocyst apoptosis rates [87,117,118,119]. The positive effect of royal jelly on oocyte maturation is related to the presence of antioxidant amino acids (cysteine, lysine, and arginine) and polyphenols [117].

## 5. Honeybee Products and Reproductive Disorder Mitigation

### 5.1. Reproductive Toxicity of Pollutants and Heavy Metals

Recently, synthetic chemicals used for different industrial and agricultural purposes have been increasing environmental pollution. Exposure to these chemicals via direct contact during manufacturing and handling, consumption of polluted foods/water, or inhalation of polluted air can cause reproductive toxicity and increase the risk of subfertility/infertility.

Heavy metals such as cadmium chloride (CdCl_2_) [120], copper (Cu) [121], and aluminum chloride (Al Cl_3_) [122] have been confirmed to induce subfertility/infertility in male and female mammals. These heavy metals can exert reproductive toxicity through several mechanisms. Heavy metals can accumulate inside vital organs and gonads and can evoke oxidative and inflammatory stress, leading to negative impacts on reproductive function, hormone balance, gamete quality, and fertilizability of gametes, as well as producing teratogenic effects in fetuses [120,121,122,123,124]. The efficacy of honeybee products for alleviating heavy metal reproductive toxicity has been demonstrated. Royal jelly was shown to fix hormonal alterations, oxidative status, inflammatory response, and apoptotic cascades induced following Cd-exposure, presumably due to its potent antioxidant activity [120]. Similarly, propolis protected against the toxic effects of excess Cu [121] or Al Cl_3_ [122] on testicular tissue and semen quality traits in rats, as well as by scavenging ROS and improving testicular and blood plasma redox status.

Synthetic pesticides, such as chlorpyrifos (organophosphorus insecticide), cypermethrin, and triphenyltin, are used to control a variety of agricultural, animal farming, and indoor pests [123,124,125,126,127,128]. Such chemicals can induce direct reproductive toxicity and may also act as endocrine disruptors, producing estrogen or androgen-like effects [125] and leading to various health hazards. Several studies have confirmed the protective role of propolis against pesticide-induced reproductive toxicity in males [25,122] and females [126]. The protective role of propolis is ascribed to the antioxidant and anti-inflammatory actions of polyphenols baicalin, lucenin 2, and quercetin against neuroprotective toxicity. Moreover, the anti-inflammatory activity of alkaloids and hepato-protective role of the organosilicon compounds in propolis were proposed as protective mechanisms [125]. Interestingly, honeybee products could also be used to reduce the toxicity of naturally occurring toxins. El-Nekeety et al. [126] reported that royal jelly resulted in a significant reduction in the toxic hazards of fumonisins (mycotoxins), likely due to increased glutathione peroxidase synthesis and suppression of lipid peroxidation and free radical generation by antioxidant enzymes. The protective effects of honeybee products against chemical stress induced reproductive toxicity in mammals are summarized in Table 7.

### 5.2. Unhealthy Lifestyle and Psychological Stresses

Lifestyle factors related to individual habits and ways of life can substantially impact overall health and well-being, including reproductive health. Nutrition, weight, exercise, psychological stress, and other factors can have substantial effects on fertility (Table 8). Lifestyle factors such as cigarette smoking and drug addiction can negatively influence fertility [127]. For instance, smoke from cigarettes, household wood and coal fires, barbecue grills, and automobile exhaust can elevate levels of harmful toxins, such as nicotine [128] and benzo[a]-pyrene [129], in the bloodstream. These toxic compounds are associated with decreased sperm count and motility and an increased percentage of morphologically abnormal sperm, sperm chromatin damage, erectile dysfunction, and early pregnancy loss [130]. Studies using animal models found that the negative impacts of toxins could be mitigated by the consumption of honey [131], royal jelly [128], and propolis [129]. The positive impacts of honeybee products are related to their antioxidant and endocrine-modulating activity, as some bee products, specifically royal jelly, include sex hormones among their constituents [85]. Human exposure to harmful synthetic chemicals may also occur because of the use of chemicals in food and healthcare products, which are considered safe for humans. For example, monosodium glutamate (MSG; flavor enhancer) is used as an ingredient in various food products; however, negative impacts of MSG on male infertility (testicular hemorrhage, degeneration and alteration of sperm cell population and morphology) have been documented [132]. Similarly, sodium fluoride, a main component of toothpaste, can induce reproductive disorders [133]. Bee propolis has been identified as a suitable supplement for alleviating such negative effects [133,134].

Furthermore, psychological stress contributes to many reproductive disorders and dysfunctions. Haron et al. [135] found that administering Tualang honey (1.2 g/kg daily) to restraint-stressed pregnant rats conferred beneficial effects on reproductive parameters, such as corticosterone level and pregnancy outcome. Another study on rats exposed to prenatal restraint stress reported that impaired reproductive function in male rat offspring could be improved by feeding dams honey (1.2 g/kg, three times per day) from day 11 of pregnancy until delivery [136]. Moreover, Rajabzadeh et al. [137] reported that dissolving 0.2 mL of 5% honey in the water of rats exposed to auditory stress significantly improved fertility rates and fetus health.

## 6. Precautions and Hazards

As shown through the review, honeybee products have several beneficial effects on the reproductive health of mammals. However, adverse effects of honeybee products on reproduction in mammals have been also reported, specifically when they are consumed during critical periods of the reproductive cycle, such as puberty and pregnancy. The effects of royal jelly on the reproductive system of puberty male rats were investigated in vivo after daily administration of royal jelly at doses of 200, 400, and 800 mg/kg for four weeks. The high-dose royal jelly oral administration adversely affected the reproductive system by decreasing testis weight, changing testis microstructure, and increasing sperm deformity rate of the assayed rats, but the unfavorable effects were alleviated by treatment cessation [37]. The adverse effects of royal jelly were attributed to the estrogenic activity of a high dose of royal jelly. In males, the improper intake of substances with estrogenic activity can adversely affect the reproductive performance of males at different reproductive windows [138,139]. In another study, a low dose of Indonesian propolis (380 mg/kg) and a high-dose of propolis (1400 mg/kg) were provided to mice for 18 days of gestation to confirm the safety of consuming propolis during pregnancy [38]. The low dose of propolis increased weight, crown-rump length, and ossification center thickness compared with the control group. Conversely, the high-dose of propolis reduced weight, crown-rump length, and ossification center thickness and caused hypertrophy of the placenta, inhibiting fetal development. These results may be attributed to the immunomodulatory properties of propolis, as fetal resorption may occur due to rejection via the immune system pathway. Increased macrophage activity in the endometrium during pregnancy leads to increased production of NO and TNF-α, which are toxic to embryo development. Further, it was found that colony-stimulating factor-1 (CSF-1) increased resorption in pregnant mice; CSF-1 plays an important role in the differentiation of macrophages. The anticancer activity of propolis was also proposed as an underlying mechanism of fetal growth retardation in high-dose groups. It is known that most anticancer agents are teratogens, and vice versa. Thus, some natural products that have anticancer effects may also be teratogenic. However, teratogenic effects were not observed after the administration of low doses [38]. Therefore, honeybee product dose should be carefully considered, as high concentrations of royal jelly and propolis can negatively impact sperm cell quality and fetal development [104].

## 7. Conclusions

The study of the unique chemical composition of honeybee products and their effects on the reproductive performance of mammals provides opportunities to detect pharmaceutical molecules for safe reproductive health manipulation. The biological activities (e.g., antioxidant, antimicrobial, antiviral, anti-inflammatory, immunomodulatory, antifungal, wound-healing, and cardio-protective) of honeybee products were ascribed to the phenolic compound and terpenoid constituents. Honeybee products have been demonstrated to improve libido, erectile function, spermatogenesis, epididymal sperm count, and normal sperm percentage, as well as to reduce sperm head and tail abnormalities and chromatin damage in many mammalian species. The benefits of using bee honey as a natural cryoprotectant agent in semen cryopreservation and liquid storage media were also reported. Moreover, honeybee products can improve female reproductive performance and milk production and fetal immune status and growth performance. Given the chemical constituents of honeybee products, inclusion in in vitro maturation (IVM) or in vitro fertilization (IVF) media may produce positive effects on oocyte maturation, fertilizability, and division and embryo development. Many studies have also confirmed the efficacy of honeybee products for alleviating the reproductive toxicity of chemical contaminants and pollutants. However, high-dose oral administration of honeybee products may adversely affect the reproductive system; thus, doses should be carefully considered when administering such products. Overall, the bioactive components of honeybee products, when wisely used, can provide a natural approach for improving the reproductive performance and health of mammals.

## Figures and Tables

**Table 1:** The major species of honeybees and their geographical distribution.

Honeybee Species (Scientific Name)	Common Name/Domestication	Region
*A. mellifera*	Western honeybee/domesticated	Old World Europe, Eastern Mediterranean, and Africa
*A. cerana*	Asiatic honeybee/domesticated	Southern, Southeastern, and Eastern Asia
*A. dorsata*	Giant honeybee/wild	Southern and Southeastern Asia
*A.florea*	Red dwarf honeybee/wild	Southern and Southeastern Asia
*A. andreniformis*	Black dwarf honeybee/wild	Southeastern Asia
*A. laboriosa*	Himalayan giant honey bee/wild	Asia (Himalayas, mountainous regions of Bhutan and China, India, and Vietnam)
*A. koschevnikovi*	Koschevnikov’s honeybee/wild	Asia (Malaysian and Indonesian Borneo)
*A. nigrocincta*	Philippine honeybeelwild	Asia (The Philippine island of Mindanao and Indonesian islands of Sangihe and Sulawesi)

**Table 2:** Chemical composition of different honeybee products.

Honeybee Product	Main Component	Individual Components
Honey [8,43,44]	Sugars	Disaccharides (maltose and sucrose), monosaccharides (glucose and fructose), and oligosaccharides (maltotriose and panose)
	Amino acids	Arginine, glutamic, histidine, lysine, phenylalanine, proline, tyrosine, and valine
	Organic acids	Gluconic, acetic, butyric, citric, formic, lactic, malic, pyroglutamic, and succinic acids
	Vitamins	B_1_, B_2_, B_3_, B_5_, B_6_, B_8_, B_9_, B_12_, and C
	Minerals	Ca, Cu, Fe, K, Mg, Mn, Na, P, S, and Zn
	Enzymes	Amylase, glucose oxidase, and sucrase (α-glucosidase)
	Phenolic compounds	Acacetin, apigenin, benzoic acids, caffeic, chlorogenic, chrysin, ellagic, ferulic, fisetin, galangin, gallic, genistein, 3-hydroxybenzoic, 4-hydroxybenzoic, hesperetin, kaempferol, luteolin, myricetin, naringenin, p-coumaric, pinobanksin, pinocembrin, quercetin, rosmarinic, syringic, and vanillic
Royal jelly [15,45,46,47,48,49]	Sugars	Glucose and fructose
	Proteins/peptides	Apisimin, major royal jelly proteins (MRJPs 1–9), jelleines, and royalisin
	Fatty acids (carboxylic acids)	10-hydroxy-2-decenoic acid, 10-hydroxydecanoic acid, 4-hydroxyperoxy-2-decenoic acid ethyl ester, and decanoic acid (sebacic acid)
	Vitamins	B_1_, B_2_,B_3_, B_5_, B_6_, B_9_, and β-carotene
	Minerals	Cu, Fe, K, Mg, and Zn
	Hormones	Estradiol, progesterone, prolactin, and testosterone
	Phenolic compounds	Apigenin, caffeic acid, gallic acid, 4-hydroxy-3-methoxyphenylethanol, 4-hydroxybenzoic acid-methyl ester, 4-hydroxybenzoic acid, 4-hydroxyhydrocinnamic acid, hydroquinone, isorhamnetin, kaempferol, luteolin, methyl salicylate, 2-methoxy-p-cresol, 2-methoxyphenol, naringenin, p-coumaric, pinobanksin, pyrocatechol, quercetin, and rutin
Propolis [50,51,58]	Sugars	Fructose, glucose, and sucrose
	Fatty acids	Arachidonic acid, cis-13,16-docosadienoic acid, cis-11,14,17-eicosatrienoic acid, cis-5,8,11,14,17-eicosapentaenoic acid, eicosadienoic acid, elaidic acid, heneicosylic acid, linoleic acid, oleic acid, palmitic acid, palmitoleic acid, and α- and γ- linoleic acids
	Terpenoids	Clerodane diterpenoids, farnesol, isocupressic acid, labdane, and 13-symphyoreticulic acid
	Phenolic compounds	2,2,dimethyl-8-prenylchromene, apigenin, benzofuran, caffeic acid and its derivatives, chrysin, cinnamic acid and its derivatives, ferulic acid, galangin, kaempferol and its derivatives, naringenin, p-coumaric acid, pinobanksin, pinocembrin, pinostrobin, quercetin, and tectochrysin
Bee venom [14,15,16,17,18,19,20,21,22]	Sugars	Fructose and glucose
	Proteins/peptides/amines	Adolapin, apamin, dopamine, histamine, mast cell degranulating peptide, melittin, noradrenaline, procamine, protease inhibitors, secapin, and tertiapin
	Minerals	Ca, Mg, and P
	Enzymes	Glucosidase, hyaluronidase acid, lysophospholipase, phospholipase A2, phospholipase B, and phosphomonoesterase
Bee pollen [58,59,60,68]	Sugars	Fructose, glucose, and sucrose
	Fatty acids	Linoleic acid and linolenic acid
	Phenolic compounds	Apigenin, caffeic acid, catechin, delphinidin, ferulic acid, galangin, gallic acid, isorhamnetin, luteolin, naringenin, p-coumaric acid, protocatechuic acid, quercetin, rutin, and syringic acid
Beeswax [66]	Fatty acids	15,hydroxypalmitic acid, oleic acid, and palmitic acid
	Vitamins	A, B_1_, B_6_, choline, and rutin
	Minerals	Ca, Cu, Fe, K, Mn, Na, P, and Zn
	Hydrocarbons	Hentriacontane, heptacosane, nonacosane, pentacosane, and tricosane
	complex wax esters	15-hydroxypalmitic acid and diols
Bee bread [67]	Sugars	Monosaccharides (glucose and fructose) and disaccharides (erlose, maltose, turanose, and trehalose)
	Fatty acids	Arachidic acid, arachidonic acid, docosahexaenoic acid, eicosapentaenoic acid, linoleic acid, myristic acid, oleic acid, palmitic acid, and α-linolenic acid
	Vitamins	B_1_, B_2_, B_3_,B_5_, B_6_, B_9_, C, E (α, β, and γ-tocopherol), and K
	Minerals	Ca, Cu, Fe, K, Mg, P, Se, and Zn
	Enzymes	Amylase, phosphatases, and saccharase
	Phenolic compounds	Apigenin, chrysin, kaempferol, naringin, p-coumaric acid, quercetin, and rutin
	Sugars	Lactulose, melizitoze, neo, trehalose, raffinose, sucrose, trehalose, (α- and β-) isomaltose, and (α- and β-) maltose
Drone brood [63,69]	Amino acids	Alanine, asparagine, aspartic acid, glutamine, glycine, histidine homoserine, isoleucine, leucine, lysine, methionine, phenylalanine, proline, pyroglutamic acid, sarcosine, serine, threonine, tyrosine, valine, and γ-aminobutyric acid
	Fatty acids	3-ethylglutaconic acid, 2-hydroxyglutaric acid, 3-hydroxybutyric acid, adipic acid, fumaric acid, glyceric acid, lactic acid, malic acid, octadecanoic acid, oleic acid, palmitic acid, and succinic acid
	N, containing compounds	Adenosine- tetra-TMS, uracil, urea, uric acid, and uridine
	P, containing compounds	Glucopyranosyl phosphate, phosphoric acid, and (α- and β-) glycerylphosphate
	Sterols	Avenasterol, campesterol, and β-sitosterol

Minerals (Ca: calcium, Cu: copper, Fe: iron, K: potassium, Mg: magnesium, Mn: manganese, N: nitrogen, Na: sodium, P: phosphorus, S: sulphur, and Zn: zinc) and vitamins (A: retinoids, B_1:_ thiamine, B_2:_ riboflavin, B_3_: nicotinic acid, B_5_: pantothenic acid, B_6_: pyridoxine, B_8_: biotin, B_9_: folic acid, B_12_: cyanocobalamin, C: ascorbic acid, and E: tocopherol).

**Table 3:** Summary of some recent studies on the effects of different honeybee products on reproductive performance of males in different mammalian species.

Animal Species	Treatment/Honeybee Product	Result	Suggested Mode of Action
Rats [16]	1 mL/100 g BW of honey for 65 days	Improved semen quality (sperm count, sperm motility, and sperm morphology)	Improving spermatogenesis and steroidogenesis Providing energy source for sperm cells by increasing sorbitol dehydrogenase activity Protecting germ cells against oxidative stress due to antioxidant activity of pinocembrin, pinostrobin, vitamins, and glucose oxidase Improving spermatogenic cells proliferation
Rats [31]	Drinking 5% solution of Palestinian honey for 20 days	Increased relative weight of epididymis and epididymal sperm count Increased SDH and reduced LDH activities	
Rats [32]	0, 0.2, 1.2, and 2.4 g/kg BW/day of Malaysian honey for 4 weeks	At 1.2 g/kg BW/day, increased epididymal sperm count	
Rats [33]	1.0 mL/100 g BW/day of Gelam honey for 60 days	Improved spermatogenic cells and sperm count and percentage of normal sperm and decreased abnormal sperm	
Rats [19]	2.5%, 5%, 7.5%, 10%, and 12.5% of propolis extract for 18 days	At 10%, increased T level, spermatogenesis, and sperm motility	Antiestrogen effect due to flavonoids (kaempferol, quercetin, and isorhamnetin) content
Rabbits [20]	0, 0.25, 0.50, 0.75, 1.0, and 1.25 g/kg diet of propolis for 94 days	At 1.25 g/kg diet, improved semen quality	Antioxidant properties of the flavonoids
Rabbits [21]	0.5 and 1 g/animal/day of Egyptian propolis for 6 weeks during summer months	At 0.5 g/animal/day, improved T level, semen volume, sperm motility, morphology, and viability traits, as well as seminal plasma fructose levels	Antioxidant and anti-inflammatory effects
Rabbits [14]	150 mg/kg diet of vitamin E or propolis for 10 consecutive weeks during summer months	Both treatments improved libido, sperm count and viability, seminal plasma fructose and total protein level, and improved plasma antioxidant activity (TAC and MDA) and T level	Antioxidant agent providing protection against lipid peroxidation
Pre-pubertal rabbits [22]	15 mg/kg BW of propolis with/without 200 mg royal jelly + 0.25 mL bee honey	Both treatments accelerated age of puberty and improved libido, ejaculate volume, sperm concentration, sperm motility and morphology, seminal plasma fructose levels, blood plasma T levels, and fertility	Antioxidant agent
Rats [23]	3, 6, and 10 mg/kg BW/day of Brazilian green propolis extract for 56 days	At 6 mg/kg/day, increased sperm production and greater epithelium height of the epididymis initial segment	Protecting sperm DNA fragmentation from thiobarbituric acid-reactive substances
Rats [27]	1 g/kg BW of royal jelly with or without hydrogen peroxide (0.5%) in drinking water for one month	Royal jelly with or without hydrogen peroxide increased testicular weight and the body of epididymis, sperm count, T and glutathione levels, and decreased sperm deformity percentage	Stimulating gonadotropins secretion owing to acetylcholine stimulation Improving spermatogenesis by zinc L-arginine and carnitine amino acid Antioxidant activity of vitamin C, vitamin E, and arginine
Pups [28]	125, 250, and 500 mg/day/kg diet of royal jelly proteins	At 125 and 250 mg/day/kg diet, improved development of testis at neonate period until pubescent, testis weight, diameter of seminiferous tubule, and height of seminiferous epithelium	Antioxidant, antibacterial, anti-inflammatory activities of amino acids and 10- hydroxy -2- decanoic fatty acid Spermatogenesis stimulating effects of l-arginine and carnitine amino acids
Rabbits [29]	0, 50, 100, and 150 mg/kg BW of Chinese royal jelly	All doses increased total sperm output	Decreasing cellular damage, lipids peroxidation, and DNA fragmentation due to antioxidant activity
Pre-pubertal rabbits [30]	0.25 mL honey, 200 mg royal jelly, and 200 mg royal jelly + 0.25 mL honey	All treatments accelerated puberty, improved ejaculate volume and sperm quality, seminal plasma fructose concentration, T and cholesterol levels, conception rate, and litter size	Antioxidant agent
Sheep [35]	10, 15, and 20 mg/kg diet of apistimul preparation (drone brood) for 95 days	Improved the qualitative and quantitative characteristics of the ejaculate Increased the amount of hemoglobin and erythrocytes in the blood	Positive effects of sex hormones and sulfhydryl groups in the drone brood on semen quality variables
Pigs (junior boars) [34]	Parenteral injection with alcohol extracts of the drone brood	Increasing the weight of the seminal glands and epididymis After injection of the drone brood homogenate extract, 33.3% of boars recovered their sexual function in 30 days, while 83.3% of breeders recovered in 2 months	
Rabbits [36]	Injection of 0.1, 0.2, and 0.3 mg/rabbit of bee venom twice weekly for 20 weeks	At all levels, improved libido, sperm concentration, viability T level, and blood biochemical (total protein, albumin, and glucose), as well as antioxidant markers (TAC, GST, and GSH)	Growth promoter Anti-inflammatory, anti-microbial, and antioxidant activity
Rabbits [18]	0, 100, 200, and 300 mg of bee pollen/kg BW	At 200 mg/kg BW, improved semen quality, fertility rate, and blood biochemicals profile	Antioxidant activity

BW: body weight, DNA: deoxyribonucleic acid, GSH: glutathione, GST: glutathione-S-transferase, g: gram, kDa: kilo dalton, kg: kilogram, LDH: lactate dehydrogenase, MDA: malondialdehyde, mg: milligram, ml: milli, SDH: sorbitol dehydrogenase, SOD: superoxide dismutase, T: testosterone, and TAC: total antioxidant capacity.

**Table 4:** Effect of bee products on reproductive performance of females of different mammalian species.

Animal Species	Treatment/Honeybee Product	Result	Suggested Mode of Action
Ovariectomized rats a model for menopausal symptoms in women [76]	0.2, 1.0, and 2.0 g/kg/day of Tualang honey for 2 weeks	At all levels, increased uterus weight and vaginal epithelium thickness, restored the morphology of the tibia bones, and decreased P_4_ and E_2_ levels	Estrogenic activity of flavonoids (kaempferol and quercetin) Antioxidant activity
Rabbits and offspring [82]	150 and 300 mg/kg diet/day of bee pollen and/or propolis (Bp + Pro) three times a week along eight parities	At 150 or 300 mg of Bp + Pro alone or together, improved reproductive performance, milk production, litter size viability and weights, and immune status of does	Antibacterial, antiviral, antiparasitic, anti-inflammatory, immunomodulatory, and antioxidant properties. Improving gastrointestinal microflora homeostasis High nutritional value (polyunsaturated fatty acids, mineral, vitamins, and amino acids)
Rabbits [83]	0.2 g/kg BW of bee pollen and/or propolis compared with 35 mg/kg BW prebiotic (inulin and/or MOS)	MOS and bee pollen with or without propolis treatments increased P_4_ and E_2_ levels and fertility Bp with propolis increased litter size and number of live kits at birth	Antioxidant activity of flavonoids and carotenoids Improving nutritional status Presence of natural growth promoters Improving lipid metabolism owing to the action of linolenic fatty acid in bee pollen Improving sex steroidogenesis
Rats [84]	3 and 5 g/kg feed mixture of rape seed bee pollen	At 5 k/kg, decreased IGF-I release by rat ovarian fragments, and increased P_4_ and E_2_ secretion	Regulating ovarian steroidogenesis Regulating antiapoptotic and pro-proliferating pathways
Rabbits and offspring [85]	100, 200, and 300 mg/kg BW of bee pollen extract for 1 week before and after mating	At 200 mg/kg BW, increased E_2,_ conception rate, litter size and survival at birth, and milk yield and body weight of kits	Improving nutritional status Antioxidant activity of flavonoids, carotenoids, and phenolic constituents Improving lipid metabolism
Polycystic ovarian syndrome animal model using female rats [46]	200 and 400 mg/kg/day of royal jelly for 4 weeks	Increased FSH and TAC levels and decreased LH, E_2_, T and MDA levels, and the size of cystic follicles	Antioxidant properties of caffeic acid and anti-inflammatory properties of sebacic acid Anti-androgenic effect Modulating estrogenic activity by 10-HDA and HDAA
Sheep [80]	250 mg/ewe of royal jelly during 12 days of estrous synchronization	Induced estrus and increased first service conception rate	Gonadotropin hormones-like action
Sheep [77]	400 mg/ewe of royal jelly during the period of CIDR-treatment	Improved pregnancy and lambing rates	Gonadotropin hormones-like action
Rats [81]	Intraperitoneal treatment with 100, 200, and 400 mg/kg BW/day royal jelly for 14 days	At all levels, increased uterine and ovarian weights and the serum levels of P_4_ and E_2_, and number of mature follicles and corpora lutea	Royalactinn, a 57 kDa protein, acts as a growth promoter Amino acid roles in tissue synthesis and body growth Steriodogenesis stimulating effects, particularly progesterone Antioxidant activity

W: body weight, Bp: bee pollen, CIDR: progesterone (P_4_)-releasing devices, eCG: equine chorionic gonadotropin, E_2_: estradiol, FSH: follicle stimulating hormone g: gram, HDAA: hydroxy-decenoic acid, 10-HAD: 10-hydroxy-2-decenoic acid, kDa: kilo Dalton, kg: kilogram, LH: luteinizing hormone, MDA: malondialdehyde, MOS: mannan-oligosaccharides, mg: milligram, ml: milli, P_4_: progesterone, Pro: propolis, T: testosterone, TAC: total antioxidant capacity.

**Table 5:** Effect of honeybee products on assisted reproductive techniques (ARTs) in males.

Animal Species	Treatment/Honeybee Product	Result	Suggested Mode of Action
Humans [90]	0%, 5%, or 10% of honey as cryoprotectant	At 10%, enhanced post-thawing semen quality	Antioxidant and antimicrobial activity of organic acids, vitamins, and phenolic acids Cryo-protectant properties of glucose, fructose, and sucrose, minimizing formation of ice crystals inside the sperm cytoplasm Providing sperm cells with energy (sugars)
Buffalos [94]	1%, 2%, 3%, 4%, and 5% of honey to cooled and frozen tris-based semen extenders	At 1%, improved motility of cooled sperm cells At 2%, improved motility of post-thawing sperm cells	
Boars [99]	1.0%, 1.5%, and 2.0% of honey to liquid storage semen extender	At 1.5 and 2.0%, improved motility and viability of sperm cells	
Bulls [92]	1%, 2.5%, 5%, 10%, and 15% of honey to frozen semen extender	At 1%, increased sperm motility and viability post-chilled, and post-thawed frozen	
Bulls [93]	2.5%, 5%, and 10% of honey to frozen semen extender	At 2.5% honey, improved post cryopreservation semen quality (progressive motility, livability, and normal morphology)	
Rabbits [96]	0%, 1%, 3%, and 5% of honey to cooled semen extender (cooling at 4 °C for 72 h)	At all concentrations, improved semen quality, acrosomal integrity, and antioxidant status	
Sheep [103]	0%, 0.5%, 1%, 1.5%, and 2% of royal jelly to liquid storage semen extender	At 0.5 and 1%, improved sperm kinetics and plasma membrane functionality	The cell membrane protective role of essential amino acids and 10-hydroxy-2-decenoic acid Anti-inflammatory and antioxidant activities
Buffaloes [105]	0%, 0.05%, 0.1%, 0.2%, 0.3%, and 0.4% of royal jelly to semen freezing extender	At 0.1%, improved sperm viability, plasma membrane, and acrosome integrity	
Rabbits [111]	0.8, 1.2, 1.6, and 2.0 mg of propolis ethanolic extract/5 mL of frozen semen extender	At 1.2 and 1.6 mg, maintained semen characteristics	Protective role of chlorogenic acid against lipid peroxidation Antioxidant activity of phenolic compounds such as rosmarinic acid, myricetin, Kaempferol, and apeginin-7-glucoside Antibacterial, antiviral, anti-inflammatory, and antioxidant properties of volatiles oil
Sheep [113]	400 and 600 µl of propolis powder/propolis glue compared with synthetic antibiotic	Both treatments increased sperm motility and normality and acrosome integrity Decreased ALT, AST, ALP, and LDH enzymes and bacterial count	

ALT: alanine aminotransferase, AST: aspartate aminotransferase, ALP: alkaline phosphatase, h: hours, LDH: lactate dehydrogenase, µl: microliter, mL: milli, TAC: total antioxidant capacity.

**Table 6:** Effect of honeybee products on assisted reproductive techniques (ARTs) in females.

Animal Species.	Treatment/Honeybee Product	Result	Suggested Mode of Action
Sheep [119]	Grade A and B oocytes cultured for 24 h in IVM supplemented with 0, 5, 10, and 20% honey	At 5%, improved percentage of metaphase II stage oocytes and glutathione concentration values	Antioxidant activity and gene expression modulatory effect
Goats [117]	2.5, 5, and 10 mg/mL of royal jelly to oocyte IVM media	At 5 mg/mL, increased percentage of blastocysts and decreased the apoptotic cells per blastocyst At 5 and 10 mg/mL, expression profile of Bax and p53 was down-regulated, while Bcl-2 was up-regulated	The positive effect of essential amino acids (cystine, lysine, and arginine), sugars (fructose, glucose, and sucrose), vitamins (A, B_5_, C, D, and E), and lipids Estrogenic activity of fatty acids and sterols
Sheep [118]	0, 2.5, 5, and 10 mg/mL of royal jelly to oocyte IVM media	At 10 mg/mL, increased percentage of metaphase II stage oocytes, intracellular GSH content, fertilization, blastocyst rate, and expression of PFK in liver and muscles and G6PDH genes in cumulus cells	

Bax: Bcl2-associated X protein, Bcl-2: B-cell leukaemia/lymphoma gene-2, COCs: cumulus–oocyte complexes, caspase-3: intracellular peptides associated apoptosis, G6PDH: glucose 6-phosphate dehydrogenase, IVM: in vitro maturation, mg: milligram, mL: milli, P_53_: apoptotic-induced, PFK: phosphofructokinase.

**Table 7:** Protective effects of honeybee products against chemical stresses-induced reproductive toxicity in mammals.

Animal Species/Chemical Stress	Treatment/Honeybee Product	Result	Suggested Mode of Action
Rats/Copper [121]	100 mg/kg BW/day of propolis ethanolic extract	Improved sperm quality and antioxidant status	Antioxidant activity of flavonoids
Rats/Cadmium [120]	0.5 mg/L water of cadmium chloride and 400mg/kg BW of royal jelly	Maintained sperm quality, testosterone, LH, and fertility	Antioxidant activity
Rats/Aluminum chloride [122]	mg/kg BW, 1/25 L of aluminum chloride and 50 mg/kg BW of propolis for 70 days	Improved testis histopathological structure and antioxidant status of liver, kidney, and blood	Antioxidant activity trans-cinnamic, p-coumaric, caffeic, ferulic, sinapic, caffeic acid phenethyl ester, apigenin, kaempferol, quercetin, rutin, flavonol galangin
Rats/Chlorpyrifos [124]	9 mg/kg BW chlorpyrifos and 50 mg/kg BW of propolis for 70 days	Restored sperm counts, sperm cell survival, and testosterone level	Improved metabolism owing to high content of flavonoids Antioxidant effect
Rabbits/Cypermethrin [125]	50 mg/kg BW of cypermethrin and 50 mg/kg BW of propolis	Improved antioxidant status, steroidogenesis, pregnancy outcomes, and litter characteristics	Neuroprotective role of Baicalin, lucenin 2, baicalin, and quercetin against cypermethrin toxicity Antioxidant, anti-inflammatory, antimicrobial, anticancer, anti-allergic, and anti-platelet activities of luteolin Anti-inflammatory activity of alkaloids Hepato-protective role of organosilicon compounds
Rats/Cadmium [120]	0.5 ppm/L water cadmium and 400 mg/kg BW/day of royal jelly for 60 days	Restored sperm quality, testosterone, LH, and fertility	Antioxidant agent
Rats/Fumonisin [126]	200 mg/kg diet Fumonisin B-contaminated diet and 100 or 150 mg/kg BW of royal jelly	At 150 mg/kg BW, improved antioxidant status and maintained liver and kidney structure and functions	Anti-inflammatory activity of neopterin

BW: body weight, g: gram, kg: kilogram, L: liter, LH: luteinizing hormone, mg: milligram, ml: milli, ppm: part per million.

**Table 8:** Impact of honeybee products on animal reproductive disorders induced by unhealthy lifestyle and psychological stresses.

Lifestyle and Psychological Stress/Animal Species	Treatment/Honeybee Product	Result	Suggested Mode of Action
Cigarette (CS) smoke/Rats [131]	CS for 8 min three times/day and 1.2 g/kg BW/day of honey	Increased percentages of intromission, ejaculation, mating, and fertility indexes	Antioxidant activity
Nicotine/Mice [128]	0.5 and 1 mg/kg/day of nicotine and 100 mg/kg BW/day royal jelly	Improved sperm parameters and in vitro fertilization outcome as well as sperm lipid stability	Antioxidant activity
Fluoride/Rabbits [133]	10 mg/kg BW/day of sodium fluoride and 25 mg/kg BW/day of propolis for 70-day	Reduced the oxidative stress toxicity induced by fluoride in the reproductive system	Antioxidant activity
Mono-sodium glutamine/Rabbits [134]	8 mg/kg BW of mono-sodium glutamine and 50 mg/kg BW of propolis	Improved testosterone levels and semen characteristics	Antioxidant activity
Noise/Rats [137]	Exposure to noise as a natural teratogenic factor and 5% honey of solution and 75 mg/mL vitamin E	Enhanced steroidogenesis and fertility rate	Antioxidant agent Regulating of anti-apoptotic patterns evoked by noise Improve steroidogenesis
Prenatal stress/Rats [136]	Exposure to a restraint stress and 1.2 g /kg BW of honey	Honey supplementation during prenatal restraint stress alleviated teratogenic effects on male offspring	Antioxidant agent

BW: body weight, g: gram, kg: kilogram, mg: milligram, ml: milli.
